# Haematological, renal and hepatic toxicity profiles of infusional 5-fluorouracil versus capecitabine in African gastrointestinal cancer patients: a retrospective cohort study

**DOI:** 10.3332/ecancer.2026.2121

**Published:** 2026-05-07

**Authors:** Tinashe Adrian Mazhindu, Ntokozo Ndlovu, Margaret Z Borok, Kevin Grimes, Collen Masimirembwa

**Affiliations:** 1 Department of Oncology, Medical Physics and Imaging Sciences, Faculty of Medicine and Health Sciences, University of Zimbabwe, Harare, Zimbabwe; 2 African Institute of Biomedical Science and Technology, Harare, Zimbabwe; 3 Department of Internal Medicine, Faculty of Medicine and Health Sciences, University of Zimbabwe, Harare, Zimbabwe; 4 Department of Chemical and Systems Biology, Stanford University School of Medicine, Stanford University, Stanford, CA 94305, USA

**Keywords:** fluoropyrimidine toxicity, treatment side effects, cancer complication, African cancer

## Abstract

**Introduction:**

Infusional 5-fluorouracil (5-FU) or capecitabine are commonly used in the management of gastrointestinal tract. So far very few studies have evaluated non dermatological, neurological and intestinal treatment related toxicities between these two fluoropyrimidine in African patients – evaluating the healthcare burden related to hospitalisations, blood transfusions and use of granulocyte colon-stimulating factor (G-CSF) – all of which are costly and scarce in Africa.

**Methods:**

We conducted a 10-year retrospective cohort study of black African patients with gastrointestinal cancer who received either 5-FU or capecitabine-based chemotherapy – extracted and analysed data on treatment related adverse events (TRAEs) incidence, severity and management.

**Results:**

A total of 179 participants were analysed: 100 received 5-FU and 79 received capecitabine. The incidence of any TRAE was 75/100 (75%) in the 5-FU group and 51/79 (65%) in the capecitabine group relative risk (RR 0.86, 95% confidence interval (CI) 0.7–1.05; p = 0.15). Severe TRAEs occurred at similar rates: 33% for both groups (RR 0.99, 95% CI 0.7–1.52; p = 0.95). Haematological TRAEs were comparable except for neutropenia, which was less common with capecitabine (33% versus 52%, RR 0.63, 95% CI 0.43–0.91; p = 0.01). Severe anaemia occurred more frequently in the capecitabine group compared to the 5-FU group, with a RR of 2.6 (95% CI 1.02–6.55). Hypokalemia was also lower with capecitabine (6% versus 17%, RR 0.37, 95% CI 0.14–0.97; p = 0.04). Hospitalisation, G-CSF use and red blood cell transfusions were similar between groups, but treatment interruptions were less frequent with capecitabine (16% versus 50%, RR 0.33, 95% CI 0.19–0.56; p < 0.0001). Treatment completion rates were 60% for 5-FU and 54% for capecitabine (p = 0.10).

**Conclusion:**

Both infusional 5-FU and capecitabine are generally well tolerated among African patients with gastrointestinal cancer. However, capecitabine is associated with a significantly lower risk of neutropenia and hypokalemia but a higher RR of severe anaemia with overall fewer incidents of treatment interruption.

## Introduction

Fluoropyrimidines such as 5-fluorouracil (5-FU) or capecitabine are commonly employed in the management of gastrointestinal tract (GIT) cancers, either as monotherapy or in combination regimens [1]. Capecitabine is an oral pro-drug that is enzymatically converted to 5-FU *in-vivo* via the activity of the enzymes carboxylesterase, cytidine deaminase and thymidine phosphorylase, which are encoded by the CES1, CDA and TYMS genes, respectively [2]. About 80% to 90% of administered or *in-vivo* formed 5-FU is rapidly metabolised into an inactive form by dihydropyrimidine dehydrogenase (DPD), an enzyme encoded by the DPYD gene [3]. The DPD-catalysed step is the rate-limiting stage in the catabolic pathway for 5-FU. Reduced or absent expression of DPD, caused by DPYD gene polymorphism, may increase the risk of toxicity or death. Therefore, pre-emptive DPYD pharmacogenomic testing is recommended for patients who are to receive fluoropyrimidines [4]. At present, genotyping for CES1, CDA or TYMS is not recommended for patients scheduled to receive capecitabine [5]. Currently, fluoropyrimidine recommendation guidelines are primarily derived from studies involving patients of European and Asian ancestry [6].

In the past 20 years, capecitabine has been widely utilised as a substitute for 5-FU in numerous chemotherapy regimens [7–9]. Toxicity comparison studies, mostly involving Europeans, have noted differences in risk for diarrhea, hand and foot syndrome and peripheral neuropathy side effects between infusional 5-FU and capecitabine [10, 11]. Based on these observations, numerous comparative studies into GIT, dermatological and neurological side effects incidence, severity and associated factors of capecitabine have been reported [11–13]. Studies comparing African Americans and Europeans have found inconsistent results and genetic associations regarding racial and ethnic differences in the incidence and severity of dermatological and neurological side effects from capecitabine [14, 15]. Limited studies reviewed the question of 5-FU versus capecitabine in patients of African ancestry, a population that has the widest genetic diversity [16]. Similarly, studies showing capecitabine to be cost-effective compared to infusional 5-FU, due to lower total patient costs like reduced hospitalisation or central vein catheter insertion costs, were conducted in non-African settings [17]. In Africa, much like the rest of the world fluoropyrimidines are recommended in the National Comprehensive Cancer Network^®^ harmonised guidelines for sub-Saharan Africa [18, 19].

There is a lack of specific studies comparing the incidence and severity of haematological, hepatic and renal treatment-related adverse events (TRAEs) between infusional 5-FU and capecitabine specifically in African patients. The distinct toxicity profiles of 5-FU and capecitabine are attributable to differences in their administration routes, metabolic processing – capecitabine being an oral prodrug requiring *in vivo* biotransformation – and variations in tissue distribution. Research on DPYD genetic polymorphism in Africans is ongoing, but studies focusing on genetic polymorphisms in the capecitabine metabolic pathway remain limited, despite their potential impact on drug response. More investigation is needed into the less-studied toxicities of fluoropyrimidine therapy in African populations, particularly comparing 5-FU and capecitabine, as well as evaluating th﻿e healthcare burden related to hospitalisations, blood transfusions and use ofgranulocyte colon-stimulating factor (G-CSF) – all of which are costly and scarce in Africa.

## Materials and methods

### Study design and setting

We conducted a retrospective cohort study of patients treated for GIT cancers from 2013 to 2022 at the Parirenyatwa Group of Hospitals, Radiotherapy & Oncology Centre in Harare, Zimbabwe.

### Inclusion and exclusion criteria

Adult black Africans over 18 years of age with confirmed esophageal, gastric, gall bladder, biliary tree, pancreatic, colon, rectal or anal cancer who received at least one cycle of infusional 5-FU or capecitabine as first line (or, if second line, after more than 6 months from the previous treatment) with adequate laboratory results for study endpoints were included. Exclusion criteria were incomplete records, receiving second-line therapy within 6 months or documented, multiple cancer diagnoses.

### Data collection

Demographic and clinicopathologic information, treatment details and laboratory results obtained prior to and during therapy were systematically collected using an enhanced data collection tool. Haematological adverse events were assessed by extracting data on haemoglobin (Hb) levels, as well as leucocyte, neutrophil, lymphocyte and platelet counts, both before and during treatments. For hepatic adverse events, parameters such as alanine aminotransferase (ALT), aspartate aminotransferase (AST), alkaline phosphatase (ALP), albumin and total and direct bilirubin were extracted. Renal function was evaluated through creatinine levels and the electrolytes sodium and potassium. TRAEs were graded for occurrence and severity according to the National Cancer Institute Common Terminology Criteria for Adverse Events version 5, based on the serial laboratory findings.

### Study endpoints

The primary objective the incidence of any or severe haematological, renal and hepatic TRAEs in GIT cancer patients treated with infusional 5-FU versus capecitabine. Secondary endpoints were trends in key blood and organ function markers during treatment, rates of treatment interruption, completion/discontinuations and the frequency of hospitalisations, blood transfusions and G-CSF requirements for TRAE management. Severe TRAEs were defined as NCI CTAE ≥3 grade events.

### Statistical analysis

Patient characteristics were described using mean and range or median and interquartile range (IQR) for continuous variables, according to normality determined by the Shapiro-Wilk test. Percentages were used to present categorical variables. Incidence of TRAEs and treatment modifications were expressed as proportions with 95% confidence intervals (CIs), both overall and stratified by grade within each fluoropyrimidine group. For comparison between treatment-group we employed chi-square tests for categorical data and Mann-Whitney *U*-tests for continuous data. Criteria for blood transfusion, G-CSF administration and hospitalisation (defined as additional days other than necessary for standard care) due to TRAEs were based on local guidelines: hemoglobin below 8 g/dL warranted transfusion, absolute neutrophil count below 1.5 ×10³/µL required G-CSF and hospital admission was enumerated as it was. Relative risk (RR), along with its standard error and corresponding 95% CI, was calculated using the 5-FU group as the control cohort and the capecitabine group as the exposed cohort [20]. A forest plot was generated using unadjusted calculated RR data. Statistical significance in the study was defined as a *p*-value less than 0.05.

## Results

### Participant characteristics

**Figure 1.**
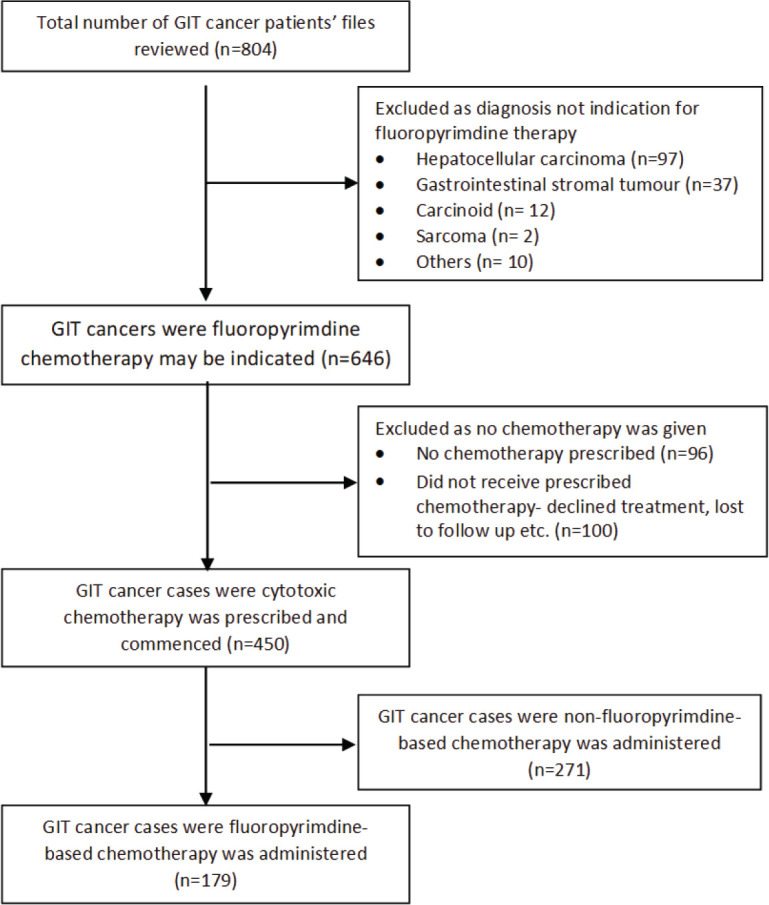
CONSORT flow chart illustrating the selection of eligible patients with gastrointestinal cancer for inclusion in the study.

Out of 804 patients diagnosed with GIT cancer during the review period, 179 fulfilled the study’s inclusion criteria (Figure 1).

Of the participants, 100 received infusional 5-FU-based chemotherapy and 79 were treated with capecitabine. Both groups had comparable demographic, lifestyle, HIV status and cancer stage at diagnosis, but differed in cancer site/type. Slightly more than half of the participants received treatment with curative intent. Of those administered 5-FU, 59% were also given platinum analogues, 41% leucovorin and 2% irinotecan as combination therapy. In the capecitabine group, 70% received oxaliplatin in combination, while 30% underwent monotherapy (Table 1).

At baseline, the haematological, renal, electrolyte and hepatic function values for study participants were similar except for serum creatinine concentration were the difference between the 5-FU group and the creatinine group were statistically significant, *p* = 0.04. However, creatinine clearance calculated using the Cockcroft-Gault formula showed insignificant differences, *p* = 0.309. An analysis of the baseline values is detailed in Supplementary Table S1.

### Treatment-related adverse events

In total, 75/100 participants (75%) in the 5-FU group experienced a TRAE, compared to 51/79 participants (65%) in the capecitabine group (RR 0.86; 95% CI, 0.70–1.05; *p* = 0.15). When considering the proportion within each group, 33% of participants experienced a severe TRAE during treatment (RR 0.99; 95% CI, 0.70–1.52; *p* = 0.95) Table 2.

**Figure 2.**
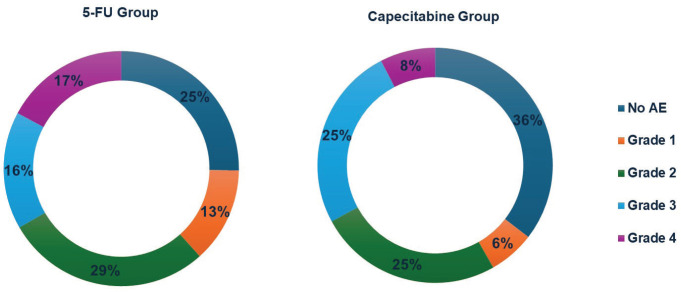
Illustrates the incidence and severity of TRAEs among gastrointestinal cancer patients treated with either 5-FU or capecitabine. The graph highlights that severe TRAEs (grades 3–4) were more frequent in the 5-FU group compared to the capecitabine group. Specifically, 52% of participants in the 5-FU group with severe TRAEs experienced grade >3 events versus 23% in the capecitabine group.

**Figure 3.**
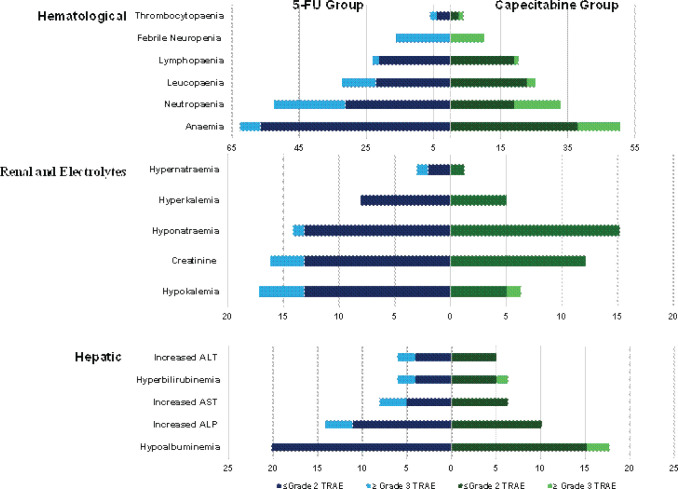
Distribution of participants experiencing any haematological, renal or hepatic TRAEs – including those classified as severe – within the 5-FU and capecitabine cohorts.

Of those with severe TRAE, 17 of 33 participants (52%) in the 5-FU group and 6 of 26 (23%) in the capecitabine group had grade >3 events. In this small subgroup analysis of participants with severe TRAE, we found a RR 0.45, 95% CI 0.21–0.97, *p* = 0.004. Figure 2 presents the distribution of TRAE grades across the treatment groups, while Figure 3 provides detailed information regarding haematological, renal and electrolyte and hepatic TRAEs.

In this cohort, rates of haematological TRAEs for 5-FU versus capecitabine were: anaemia (62% versus 51%, *p* = 0.12), neutropenia (52% versus 33%, *p* = 0.01), leucopaenia (32% versus 25%, *p* = 0.33), lymphopenia (23% versus 20%, *p* = 0.66), febrile neutropenia (16% versus 10%, *p* = 0.24) and thrombocytopenia (6% versus 4%, *p* = 0.51). A comparison of haematological TRAEs revealed that only neutropenia differed significantly between the 5-FU and capecitabine groups, with capecitabine being associated with a lower risk (RR 0.63, 95% CI 0.43–0.91). No statistically significant differences were observed for other haematological TRAEs. In a subgroup analysis among participants who experienced haematological TRAE, the rates of severe TRAE for 5-FU versus capecitabine were as follows: anaemia 10% versus 25% (*p* = 0.04); neutropenia 40% versus 42% (*p* = 0.87); leucopaenia 31% versus 10% (*p* = 0.11); lymphopenia 9% versus 6% (*p* = 0.78); and thrombocytopenia 33% versus 33% (*p* = 1.0). Severe anaemia occurred more frequently in the capecitabine group compared to the 5-FU group, with a RR of 2.6 (95% CI 1.02–6.55).

Renal and electrolyte TRAEs were similar for 5-FU and capecitabine: increased creatinine (16% versus 15%, *p* = 0.43), hyponatremia (14% versus 15%, *p* = 0.84), hyperkalemia (8% versus 5%) and hypernatremia (3% versus 1%, *p* = 0.45). Hypokalemia was less frequent with capecitabine (17% versus 6%, *p* = 0.04; RR 0.37, 95% CI 0.14–0.97). Subgroup analysis showed no significant difference in renal or electrolyte TRAEs between groups. Hepatic TRAE rates were similar between 5-FU and capecitabine groups: hypoalbuminemia (20% versus 18%, *p* = 0.70), increased (14% versus 10%, *p* = 0.44), AST (8% versus 6%, *p* = 0.77), hyperbilirubinemia (6% versus 6%, *p* = 0.94) and ALT (6% versus 5%, *p* = 0.77). No significant differences in overall any or severe TRAE risk were found between the treatments. Figure 3 below shows the distribution of any and severe TRAE. Supplementary Table S2 details all the TRAE and the corresponding RR.

### Impact of TRAEs

**Figure 4.**
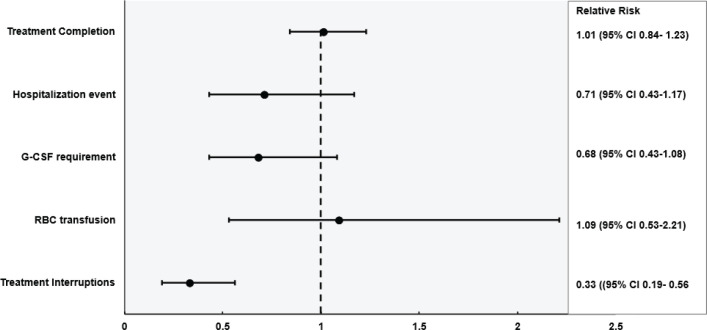
Presents a forest plot of RR and 95% CI comparing the 5-FU and capecitabine groups for treatment completion (p = 0.86), hospitalisations (p = 0.18), G-CSF use for neutropenia (p = 0.10), RBC transfusion (p = 0.82) and treatment interruption (p < 0.0001). Capecitabine-treated participants had fewer chemotherapy cycle delays than those treated with 5-FU.

A total of 714 chemotherapy cycles were reviewed across the entire cohort of 179 patients. In comparison between the 5-FU and capecitabine treatment groups, treatment interruptions – defined as at least one chemotherapy cycle delayed by more than 1 day – were observed 4 in 50 patients (50%) versus 13 patients (16%), respectively (RR 0.33, 95% CI 0.19–0.56, *p* < 0.0001). Other treatment-related challenges, including red blood cell (RBC) transfusions, indications for G-CSF use, hospitalisations for management of TRAEs, and whether the prescribed number of chemotherapy cycles was completed, showed no significant differences between the 5-FU and capecitabine groups, as illustrated in Figure 4. Supplementary Table S3 shows details on the incidence of these events and calculated risk and CIs.

Not all participants who had an indication for RBC transfusion or G-CSF administration received these treatments; documentation shows that only 50% of both groups were given the prescribed transfusion or G-CSF therapy. The overall treatment completion rate was 60% (SD ± 34.63) for the 5-FU group and 54% (SD ± 36.23) for the capecitabine group (*p* = 0.10) and both cohorts had no recorded dose reduction.

## Discussion

This study demonstrates that there is no significant difference in the overall risk of TRAEs or severe TRAE between gastrointestinal cancer patients treated with 5-FU and those receiving capecitabine. However, among patients who experienced severe TRAE, those on 5-FU exhibited a higher tendency for grade 4 TRAE. Review of system-specific TRAE revealed that capecitabine was associated with a reduced risk of neutropenia and hypokalemia within haematology parameters, while hepatic TRAE risks were comparable between groups. The management of TRAE – including hospitalisation rates, RBC transfusion needs, indications for G-CSF administration and treatment completion – was similar across both cohorts. Notably, patients receiving capecitabine had a lower risk of treatment interruption compared to those on infusional 5-FU.

The incidence of haematological TRAE among black African patients in this study matches findings from both clinical trials and real-world data [21–23]. Among haematological TRAE, anaemia was the most common. The incidence of anaemia in patients with GIT cancers is higher than in those with non-GIT cancers, which may be related to factors such as feeding difficulties, micronutrient deficiencies, malabsorption and advanced stage of cancer at diagnosis [24, 25]. The capecitabine group had significantly lower neutropenia risk, while febrile neutropenia rates were similar to infusional 5-FU in GIT cancer patients of African ancestry. Neutropenia incidence was 7% higher than reported in our earlier study at this site, which included non-GIT cancer patients [26]. Oral capecitabine provides greater convenience and, in this study, is associated with a lower risk of neutropenia. While these factors may be relevant in treatment considerations for African patients, there are limitations to its use, such as restrictions on oral intake or higher purchase costs for certain patient groups. Implementing neutropenia risk assessment and in high-risk individuals prophylactic G-CSF administration may further enhance the haematological safety profile of fluoropyrimidine therapy, as no such adverse events were observed in this cohort [27, 28]. Febrile neutropenia carries a reported mortality rate of up to 30%. Effective risk reduction strategies, prompt detection and timely, appropriate treatment are essential for improving patient outcomes and survival rates [29–31].

Renal and electrolyte TRAE generally exhibited comparable patterns, except for hypokalemia, which presented an increased risk in the 5-FU infusion group. In patients with GIT cancers receiving cytotoxic chemotherapy, hyponatremia and hypokalemia may occur because of various factors such as vomiting, diarrhea or the direct toxic effects of platinum-based agents. Cisplatin, known for its nephrotoxic potential, was administered alongside 5-FU in one-third of participants in the 5-FU cohort within this study. No participants were treated concurrently with both cisplatin and capecitabine. Cisplatin induces systemic hypomagnesemia by reducing magnesium reabsorption in the nephron’s loop of Henle and distal tubule, resulting in associated hypokalemia [32]. In hypokalemic conditions, muscle dysfunction can lead to cardiac arrhythmias and reduced intestinal peristalsis, which may further negatively affect patients’ nutritional and hydration status [33, 34]. The mechanism behind cisplatin-induced hyponatremia remains unclear. Acute kidney injury can occur due to factors such as the syndrome of inappropriate antidiuretic hormone secretion and renal salt wasting syndrome. In this study, the 5-FU group did not, however, experience a higher risk of hyponatremia [35]. In the context of this study, the improved hypokalemia outcome in the capecitabine group may be the result from combination therapy and might not persist if capecitabine is used with cisplatin instead of oxaliplatin.

All the elements analysed for chemotherapy-induced hepatic toxicity in this study showed a similar low risk between patients in infusional 5-FU and capecitabine. Both these observations between these two treatment regimens are consistent with the reported trend in previous clinical trials and real world studies [36, 37].

These findings highlight the advantages of using oral fluoropyrimidine therapy over infusional options for African patients. In resource-limited settings, an effective cancer treatment like capecitabine – which needs less routine hospitalisation and does not increase requirements for more supportive care in the form of RBC transfusion, G-CSF or TRAE hospitalisation – offers higher treatment completion rates and fewer interruptions. This is particularly important as the study highlights limited access to G-CSF and transfusions for cancer patients. While the exact cause remains unclear, high costs and low health insurance coverage may contribute and warrant further investigation [38–41].

Current pharmacogenomic testing guidelines for both fluoropyrimidine drugs recommend pre-emptive DPYD testing [3, 42]. However, there has been limited research into polymorphisms affecting the biotransformation of capecitabine to 5-FU within African populations [43]. The findings of this study indicate that, while further investigation is warranted, capecitabine appears to be well tolerated relative to 5-FU infusion. Additionally, observed differences may be attributable to variations in administration routes, impact of diet and adherence, factors that should ideally be evaluated in a prospective study.

This study had several limitations, including a limited sample size, a heterogeneous patient population, an inability to ascertain the cause for treatment completion failures – all of which affect the generalisability of the results and the inability to assess capecitabine adherence. Additionally, local G-CSF and RBC transfusion guidelines were used, which may differ across various settings, making the conclusion subject to setting-specific conclusions. The study was also conducted at a single institution. The research only reviewed haematological, renal and hepatic TRAE, limiting full assessment of the differences between the two fluoropyrimidines; however, assessing these TRAE retrospectively was viewed to be more objective because it depended purely on objective laboratory measurements allowing for interna validity. Nonetheless, this observational study on gastrointestinal cancer toxicity in an African context, comparing infusional 5-FU with capecitabine and evaluating supportive care requirements, provides further data relevant to the field and it was conducted at single institution but the largest of just two centres in the country and only objective measurements of toxicity were used, i.e., laboratory reports.

## Conclusion

Both infusional 5-FU and capecitabine are generally well tolerated among African patients with gastrointestinal cancer. However, capecitabine is associated with a significantly lower risk of neutropenia and hypokalemia but higher RR of severe anaemia with overall fewer incidents of treatment interruption.

## Conflicts of interest

No conflicts of interest.

## Funding

Support for this work was provided through grant funding from the Bill and Melinda Gates Foundation (BMGF) (Grant Number ID INV-036801); study protocol design was partially supported by the National Center For Advancing Translational Sciences of the National Institutes of Health under Award Number UL1TR003142 through the Data Studio consultation service at the Stanford University and an award from the American Association for Cancer Research (Grant Number 24-15-75-MAZH).

## Ethical approval and consent to participate

All elements of the study were performed in accordance with the Declaration of Helsinki and the relevant regulations and laws governing research in Zimbabwe. No direct patient contact or intervention were made during this study. The study was approved by University of Zimbabwe Faculty of Medicine & Health Sciences and Parirenyatwa Group of Hospitals Joint Research Ethics Committee (JREC/263/2023) and the Medical Research Council of Zimbabwe (MRCZ/A/3159). This was a retrospective study exempt from individual informed consent.

## Data availability

The data that support the findings of this study are available from the corresponding author upon reasonable request.

## Artificial intelligence use

Microsoft^®^ CoPilot was used to rephrase some sentences written by the authors in the manuscript. The majority of the authors are not native English speakers and this rephrasing assisted with word count reductions and grammatic correction.

## Supplementary Tables

## Figures and Tables

**Table S1.** Initial haematological, renal, electrolyte and hepatic function measurements for participants receiving fluoropyrimidine therapy for gastrointestinal cancer.

Baseline characteristic	5-FU group Median (range)	Capecitabine group Median (range)	*p*-value
Haematological			
White blood cell count	6 (2.7–17)	5.8 (2.5–21)	0.55
Neutrophil count	3.5 (1–16.5)	3.4 (0.4–16)	0.77
Lymphocyte count	1.8 (0.5–4.9)	1.7 (0.4–2.5)	0.22
Platelet count	384 (167–846)	343 (34–649)	0.05
Hb	11 (6–15.2)	11.8 (5–15)	0.45
MCV	82 (54–111)	83 (56–122)	0.61
MCH	26 (18–33.7)	26.8 (17.2–41.2)	0.59
Renal function			
Sodium	138 (129–152)	139 (128–151)	0.3
Potassium	4.1 (2.4–5.6)	4.2 (3.2–5.3)	
UREA	3.6 (1.5–10.7)	3.9 (1.1–19.1)	0.42
Creatinine	68 (5.7–144)	73 (25–254)	**0.04**
Creatinine clearance	71.2 (30.7–593)	68.6 (15.5–204)	0.31
HEPATIC FUNCTION			
Total protein	72 (47–96)	71.4 (49–88.8)	0.83
Albumin	37 (21–61)	38 (18–83)	0.26
Total bilirubin	6.9 (3–80.9)	7 (1.9–74.3)	0.34
Direct bilirubin	3 (1–77.3)	2.8 (1–18)	
ALP	94 (13–1284)	104.5 (48–1,037)	0.60
AST	21 (6–214)	20 (7–180)	0.24
ALT	18 (4–167)	15 (2–203)	0.12
GGT	42.5 (13–847)	37 (2–1,322)	0.5
ALBI score grading			0.33
	43%	58%	
	54%	40%	
	3%	2%	,

Bold- significant *p*-value <0.05

**Table S2.** Haematological, renal and hepatic TRAEs; incidence, RR and significance level for any TRAE and severe TRAE.

	**RR of any TRAE among all patients from the two-treatment group**						**RR of severe TRAE among patients who experienced any TRAE between the two-treatment group^ #^**					
	**5-FU**		**Cap^∞^**		**RR**	***p* value**	**5-FU**		**Cap^∞^**		**RR**	***p* value**
	**No TRAE 5-FU**	**Any TRAE 5-FU**	**No TRAE Cap**	**Any TRAE Cap**			**No severe TRAE 5-FU**	**Severe TRAE 5-FU**	**No severe TRAE**	**Severe TRAE**		
												
Anaemia	37	63	39	40	1.24	0.12	56	7	30	10	0.39	0.03
												
Febrile neutropenia	83	17	71	8	1.6	0.24	1	15	0	8	0.94	0.60
												
Hyperbilirubinemia	94	6	74	5	0.94	0.94	4	2	4	1	1.67	0.62
												
Hyperkalaemia	92	8	75	4	1.8	0.43	8	0	4	0	*	0.58
												
Hypernatremia	96	3	78	1	3.1	0.3	3	0	1	0	*	0.68
												
Hypoalbuminemia	80	20	65	14	1.2	0.67	20	0	12	2	0	0.35
												
Hypokalaemia	83	17	74	5	2.8	0.51	13	4	4	1	1.18	0.87
												
Hyponatraemia	85	14	67	12	0.98	0.97	13	2	12	0	*	0.96
												
Increased ALP	85	14	71	8	1.4	0.34	11	3	8	0	*	0.60
												
Increased ALT	94	6	75	4	1.2	0.77	4	2	4	0	*	0.78
												
Increased AST	92	8	74	5	1.26	0.67	5	3	5	0	*	0.51
												
Leucopaenia	67	33	59	20	1.30	0.29	22	11	18	2	3.13	0.07
												
Lymphopenia	76	24	63	16	1.18	0.55	22	2	15	1	1.39	0.78
												
Neutropenia	47	53	53	26	1.6	0.01	32	21	15	11	0.95	0.87
												
Thrombocytopenia	93	6	76	3	1.8	0.5	4	2	2	1	1	1
												
Increasing Creatinine	96	4	79	0	*	0.18	2	2	0	0	*	*

^#^Sub-analysis only includes patients who any experienced any TRAE

^∞^Cap-capecitabine

*Nil

**Table S3.** RR and 95% CI for treatment completion, hospitalisation, G-CSF use for neutropenia, RBC transfusion and treatment interruption in 5-FU versus capecitabine groups.

	**5-FU group**	**Capecitabine group**	***p* value**	**RR**	**Lower 95% CI**	**Upper 95% CI**
Treatment interruptions			<0.0001	0.33	0.19	0.56
Delayed	50(50)	13 (16)				
Never delayed	50 (50)	66 (84)				
RBC transfusion			0.82	1.09	0.53	2.21
Not indicated	86 (86)	67 (85)				
Indicated	14 (14)	12 (15)				
Received	7 (7)	6 (8)				
G-CSF requirement			0.1	0.68	0.43	1.08
Not indicated	63 (63)	59 (75)				
Indicated	37 (37)	20 (25)				
Hospitalisation event			0.18	0.71	0.43	1.17
Yes	32 (32)	18 (23)				
No	68 (68)	61 (77)				
Treatment completion			0.86	1.01	0.84	1.23
Yes	30 (30)	23 (29)				
No	70 (70)	56 (71)				

**Table 1.** Demographic and clinical profiles of the study cohort.

Characteristic	5-FU N (%) n = 100	Capecitabine N (%) n = 79	p-value
**Age (years)** **Mean (IQR)**	57.02 (48–70)	53.87 (42–66)	0.12
**Sex**			
**Males**	69 (69)	45 (57)	0.86
**Females**	31 (31)	34 (43)	
**BMI** **Median (IQR)**	22 (19–24)	23 (20–25)	0.32
**HIV status**			
**Positive**	15 (15)	12 (15)	>0.05
**Negative**	85 (85)	67 (85)	
**Cancer type**			
**Esophagus**	14 (14)	13 (16)	<0.05
**GEJ**	1 (1)	8 (10)	
**Gastric**	28 (28)	7 (9)	
**Pancreas**	2 (20	0 (0)	
**Small bowel**	0 (0)	1 (1)	
**Colon**	26 (26)	29 (37)	
**Rectum**	29 (29)	13 (13)	
**Anal**	0 (0)	8 (8)	
**Cancer group stages**			
**1**	1 (1)	0 (0)	0.19
**2**	9 (9)	17 (22)	
**3**	39 (39)	27 (34)	
**4**	47 (47)	28 (35)	
**Undocumented**	6 (6)	7 (9)	
**Treatment** i**ntent**			
**Curative**	52 (52)	43 (54)	0.09
**Palliative**	48 (48)	36 (46)	
**Fluoropyrimidine plus**			
**Cisplatin**	33 (33)		
**Carboplatin**	2 (2)		
**Leucovorin (Mayo regimen)**	39 (39)		
**Leucovorin, Oxaliplatin (FOLFOX)**	24 (24)		
**Leucovorin, Irinotecan (FOLFIRI)**	2		
**Oxaliplatin**		55 (70)	
**Monotherapy**		24 (30)	
**Total number of cycles prescribed Median range**	6 1–12	8 1–8	0.78

IQR: interquartile range; BMI: Body mass index; HIV: human immunodeficiency virus; GEJ: Gastroesophageal Junction; KPS: Karnofsky Performance Status

**Table 2.** Comparative RR of TRAE incidence and severity between 5-FU and capecitabine groups.

	5-FU group		Capecitabine group		RR	p-value
	**Number**	**Proportion**	**Number**	**Proportion**		
**No TRAE**	25	25%	28	35%		
**Any TRAE**	75	75%	51	65%	0.86 (CI 95% 0.7–1.05)	0.15
**Severe TRAE**	33	33%	26	33%	0.99 (CI 95% 0.7–1.52)	0.95
